# Disordered Decision Making: A Cognitive Framework for Apathy and Impulsivity in Huntington's Disease

**DOI:** 10.1002/mds.29013

**Published:** 2022-05-02

**Authors:** Lee‐Anne Morris, Claire O'Callaghan, Campbell Le Heron

**Affiliations:** ^1^ Department of Medicine University of Otago Christchurch New Zealand; ^2^ New Zealand Brain Research Institute Christchurch New Zealand; ^3^ Brain and Mind Centre and School of Medical Sciences, Faculty of Medicine and Health University of Sydney Sydney New South Wales Australia; ^4^ Department of Neurology Canterbury District Health Board Christchurch New Zealand

**Keywords:** Huntington's disease, apathy, impulsivity, goal‐directed behavior, cost‐benefit decision making

## Abstract

A caregiver's all‐too‐familiar narrative ‐ “He doesn't think through what he does, but mostly he does nothing.” Apathy and impulsivity, debilitating and poorly understood, commonly co‐occur in Huntington's disease (HD). HD is a neurodegenerative disease with manifestations bridging clinical neurology and psychiatry. In addition to movement and cognitive symptoms, neurobehavioral disturbances, particularly apathy and impulsivity, are prevalent features of HD, occurring early in the disease course, often worsening with disease progression, and substantially reducing quality of life. Treatments remain limited, in part because of limited mechanistic understanding of these behavioral disturbances. However, emerging work within the field of decision‐making neuroscience and beyond points to common neurobiological mechanisms underpinning these seemingly disparate problems. These insights bridge the gap between underlying disease pathology and clinical phenotype, offering new treatment strategies, novel behavioral and physiological biomarkers of HD, and deeper understanding of human behavior. In this review, we apply the neurobiological framework of cost‐benefit decision making to the problems of apathy and impulsivity in HD. Through this decision‐making lens, we develop a mechanistic model that elucidates the occurrence of these behavioral disturbances and points to potential treatment strategies and crucial research priorities. © 2022 The Authors. *Movement Disorders* published by Wiley Periodicals LLC on behalf of International Parkinson Movement Disorder Society.

In Huntington's disease (HD), behavioral impairments such as apathy and impulsivity are highly prevalent^1^, ^2^ and strongly related to functional decline^3^ and reduced health‐related quality of life.^4^ The incidence of behavioral impairments in HD is highest in those with early compared with late disease onset.^5^, ^6^, ^7^ Apathy is present in up to 70% of patients.^1^, ^8^, ^9^ Impulsive behaviors too are commonly reported in HD,^8^ with 45% of patients scoring above clinical cutoffs for impulsivity.^10^ More than 90% of caregivers report at least one “risky” behavior in people with HD, with the most common being impulsive/compulsive behaviors, adverse social behaviors, and reckless driving.^11^ Both apathy and impulsivity can be evident before onset of manifest disease. Indeed, apathy has been found to occur up to 10 years prior,^1^, ^12^ while risk‐taking behaviors can be evident in premanifest gene carriers,^11^ and worsening response inhibition has been found to correlate with proximity to diagnosis.^13^ Although apathy worsens with disease progression, suggesting it is an intrinsic feature of HD,^12^, ^14^ the trajectory of impulsivity is less well characterized, and the trajectories of co‐occurring apathy and impulsivity are unknown—an important goal for future research.

Both apathy and impulsivity can manifest in different ways in different people. A reduction in goal‐directed behavior lies at the core of apathy, and this has been proposed to occur along different dimensions, including cognitive, behavioral, emotional, and social.^15^ Impulsivity is also a nonunitary trait. Current conceptualizations make a distinction between motoric forms of impulsivity (premature responding and poor inhibition of an initiated response) and decisional impulsivity (rapid decisions with poor consideration of available evidence, intolerance to time delays for reward, and preference for risky choices).^16^ Although we do not discuss these dimensions further, it may be that behavioral apathy and decisional impulsivity are most closely aligned to the framework set forth in this review.

Historically, apathy and impulsivity have been considered to exist at opposite ends of a behavioral axis, mediated in particular by dopaminergic neuromodulatory systems.^17^, ^18^ However, recent evidence demonstrates they can co‐occur in individuals with Parkinson's disease (PD),^19^, ^20^ frontotemporal lobar degeneration,^21^, ^22^, ^23^, ^24^ Alzheimer's disease,^25^ attention deficit hyperactivity disorder,^26^ schizophrenia,^27^ and even in healthy adults.^28^, ^29^ In addition, although some people may still develop either apathy or impulsivity in isolation, at a population level they are strongly correlated (Box 1). These associations suggest that apathy and impulsivity, two distinct symptomologies, arise when a common neural system underlying goal‐directed behavior is disrupted. In this review, we suggest that the system of cost‐benefit decision making (CBDM)—broadly the integration of reward and cost information to drive behavior toward goals—is a strong mechanistic candidate. We argue that pathological changes in processing of reward and cost information, *at different phases of goal‐directed behavior*, can lead to the seemingly paradoxical manifestation of both apathetic and impulsive behavior in individuals with HD. In doing so, we also emphasize the importance of the environmental context in which decisions are made, as well as an individual's ability to precisely estimate this background reward structure, as a key driver of whether changes in reward and cost processing manifest in an apathetic or impulsive manner in a given real‐world situation.

**BOX 1:**
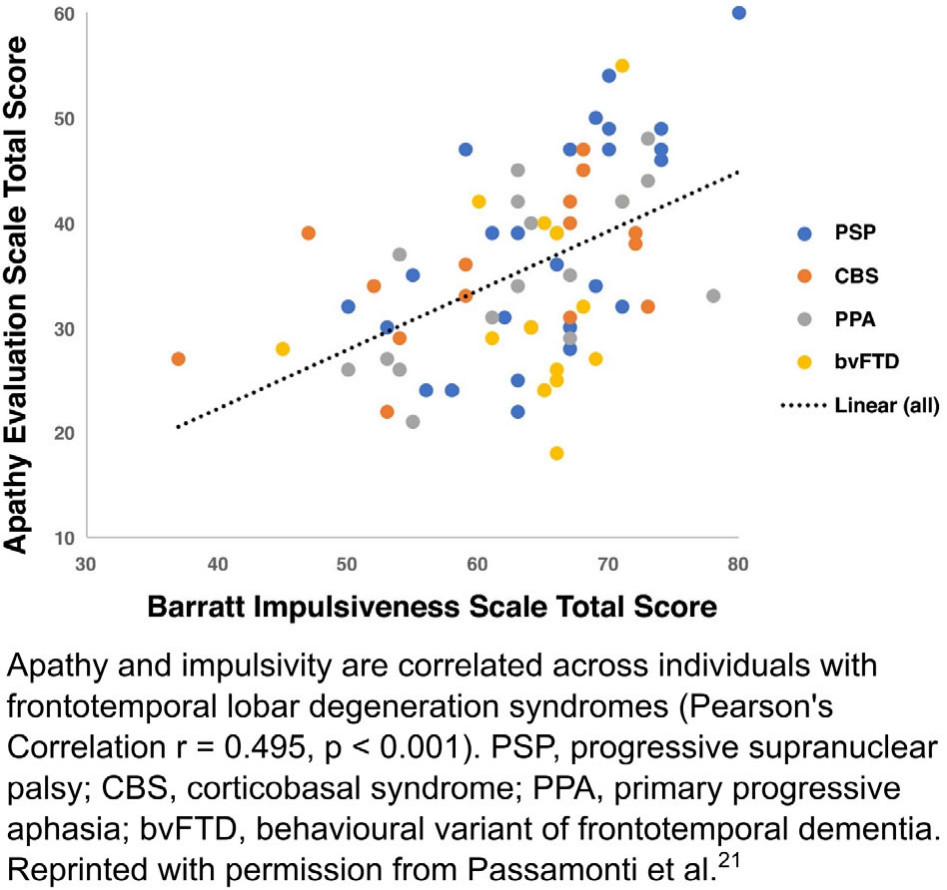
[Color figure can be viewed at wileyonlinelibrary.com]

## A Framework of Goal‐Directed Behavior With CBDM at Its Core

**FIG 1:**
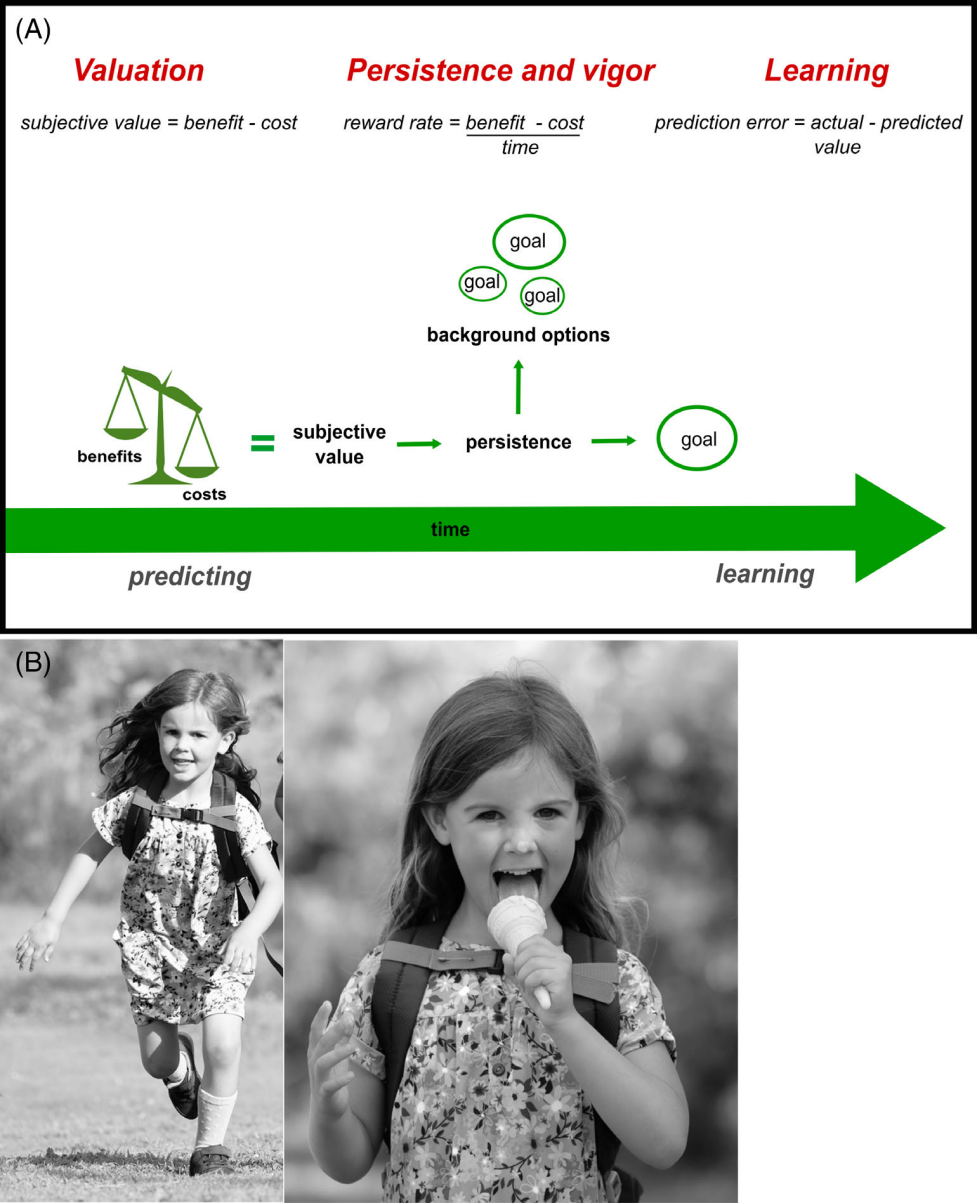
A cognitive framework for goal‐directed behavior. (**A**) Three distinct phases of cost‐benefit decision‐making (in red above) lie at the heart of the pursuit (or not) of a rewarding goal. Initially, predictions are made about the values of rewards associated with a goal and the costs that will be incurred to reach it. After behavioral activation, continued invigoration and persistence are required to attain the goal. However, an alternate option in the environment may have a higher value, in which case a behavioral switch away from the original goal may be optimal. After goal attainment (or failure of attainment), the experienced rewards and costs are compared with the predicted ones in a learning process that modifies future behavioral choices. Disruption to any of these inter‐related cognitive processes will alter goal‐directed behavior and can manifest behaviorally as apathy and/or impulsivity. (**B**) A real‐world example of goal‐directed behavior: a child decides it is worth undertaking a long (effortful) walk in return for a promised ice cream cone (reward) at the end. She must then persist with her effortful response, over time, to attain the goal. Concurrently, she evaluates the value of alternatives in her environment: if an ice cream stall was to present itself around the next corner, her initial goal may no longer be worth it. After completing the walk, and receiving her ice cream, she compares these actual costs and rewards with those she predicted at the beginning of the walk. Any difference in these values drives learning, which will inform her future decisions. [Color figure can be viewed at wileyonlinelibrary.com]

Goal‐directed behavior is a multifaceted, complex process,^35^ often encompassing a series of actions that unfold over time. It is characterized by willingness to overcome costs to obtain positive reinforcers (rewards) or to avoid negative reinforcers.^36^ CBDM describes the cognitive processes by which rewards from potential actions are weighed against the effort and time costs of those actions, to invigorate behavior toward goals. In addition to these costs, uncertainty about whether achieving a goal will result in a reward also tends to devalue potential actions (Table 1). CBDM provides an elegant framework to understand goal‐directed behavior.^37^ It has three dissociable phases that influence the production of behavior: choosing to activate behavior toward a goal, maintaining this behavior over time, and learning from the outcome of these actions, or, simply: *Is it worth it? Is it still worth it? Was it worth it?* (Fig. 1).

**TABLE 1:** Core concepts

**Subjective value:** the worth of a reward after accounting for relevant internal factors. e.g. a chocolate bar has a higher subjective value for a person when hungry compared to full. This flexible representation of value is a hallmark of goal‐directed behavior and relies particularly on the ventromedial prefrontal cortex and ventral striatum. The interested reader is referred to Levy and Glimcher^30^ for an in‐depth discussion of value.
**Costs:** (to obtain a reward): involve effort and/or temporal elements. Effort costs are directly associated with the behaviour leading to reward, and can be physical or cognitive. Time costs include delay until the rewarding outcome, but also the *opportunity cost* ‐ the value of alternatives foregone by the current behavioural goal.
**Discounting:** the decrease in value of a reward as costs increase. An immediately available reward has a higher value than the same reward available at a future time point, a phenomenon known as *temporal* or *delay discounting*. Similarly, rewards are devalued by poor odds (*probability discounting*) or increased effort requirements (*effort discounting*) to attain them.
**Sensitivity:** a measure of the separate weightings given to costs and rewards as they are integrated. Sensitivity can be quantified in computational models based on the change in behavior as rewards and costs vary, and thus estimated for individuals and groups.
**Computational models:** mathematical and algorithmic means of expressing these decision processes and variables. Whilst a discussion of computational models is beyond the scope of this review, the interested reader is referred to Pessiglione et al,^31^ Rangel and Hare,^32^ Teufel and Fletcher,^33^ and Nair et al.^34^

### Choice: Is it Worth it?

There is strong evidence from human and other animal studies that potential rewards (the outcomes of actions) and anticipated costs (to obtain the rewards) are integrated into a value signal that can drive behavior toward a goal.^38^, ^39^ Increasing costs, or the perception of costs, to obtain a reward will reduce this value signal, a phenomenon known as discounting.^40^ Similarly, insensitivity to rewards will also reduce this computed value signal. Such changes can result in a bias of choice toward it *not being worth it*, with a consequent reduction in *goal‐directed behavior* (the very definition of apathy). It may also not seem *worth it* to deliberate the values of costs and rewards; such failure to consider all relevant information manifests as reflection impulsivity.^41^ In addition, although a systematic shift in weighting of costs and rewards may manifest as relatively predictable behavioral change, integration of these decision variables is also subject to variance, or decision noise, that may be worsened by degraded connections between neural regions. This can increase variability of an individual's decisions^42^ and manifest as unpredictable behavior.

### Persistence and Vigor: Is It Still Worth It?

Because goals are usually at some physical, temporal, and/or cognitive distance from us, behaviors to reach them must be maintained across time. The (ongoing) decision to continue behavior is influenced by factors such as the value of the outcome, the probability of obtaining it, and, importantly, what else is available within the environment. In other words, what you are missing out on by continuing to pursue your current goal. Often referred to as the *opportunity cost*, this decision variable is crucial for many types of real‐world behaviors (much of which are described within the ecological framework of *foraging* ^43^, ^44^). It relies on an accurate estimate of what other options exist in your environment, an estimate that is summarized as the *environmental reward rate*, which in turn influences the vigor with which to pursue a goal. It is this background opportunity against which the current choice is repeatedly reevaluated as an agent maintains actions toward a goal, essentially asking, *Is it still worth it?* ^18^ Although this enables behavioral flexibility, if an agent cannot accurately estimate the background reward rate, the vigor with which they pursue a goal, as well as the decision to continue pursuit of it, will be suboptimal. Failure to process background reward rates may result in persistence with current low‐reward activities at the expense of shifting to a more rewarding activity. Decreased vigor as a result of a low estimation of background reward rate may also result in “giving up” on the current activity prematurely. It is notable that “lack of persistence/perseverance” is an item found in both apathy *and* impulsivity scales (eg, Barratt Impulsiveness Scale‐11, UPPS‐P Impulsive Behaviour Scale, Lille Apathy Rating Scale, Problem Behaviours Assessment), with the *context* of the abnormal behavior the crucial element in whether it appears apathetic or impulsive.

### Learning: Was It Worth It?

Behavioral processes do not simply end when an agent reaches their goal. Instead, this heralds another crucial phase: evaluating whether the rewards and costs associated with the course of action were better or worse than expected. Importantly, any discrepancy between predictions and experience is signaled by rapidly changing dopaminergic neuron activity. These “prediction errors,” extensively studied in neuroscience and psychology, update future expectations, and therefore behavior, via a process called reinforcement learning.^45^ Such a learning signal facilitates the updating of values of actions, costs, and rewards, allowing for adaptability in goal‐directed behavior. However, breakdown or biases in this process will change the way reward and cost information is evaluated, with consequent changes in behavioral production. If a person consistently finds that a goal is not as rewarding as expected (ie, a negative prediction error), over time this will manifest as reduced reward sensitivity at the choice phase of CBDM, a common finding in apathy. A similar argument can be made for loss insensitivity at the learning phase leading, over time, to apparently high‐risk/impulsive decisions at the choice phase.

## Brain Regions Subserving Goal‐Directed Behavior

**FIG 2:**
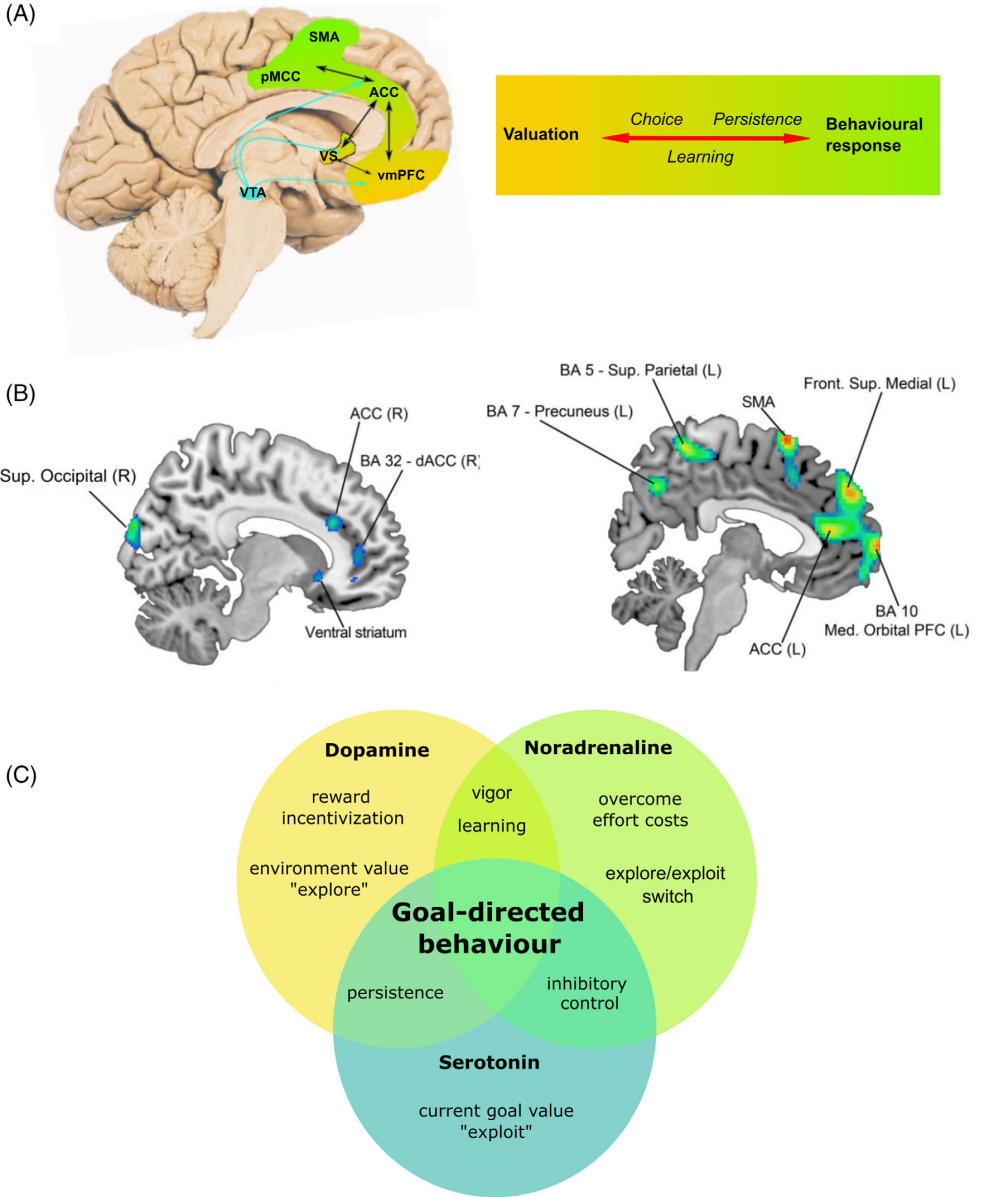
Neurobiology of goal‐directed behavior. (**A**) Brain regions forming a network that underlies all phases of goal‐directed behavior. Color shading from gold to green represents transition from valuation neural regions and cognitive processes to motor regions and processes, leading to action. (**B**) Structural (L) and metabolic (R) correlates of apathy in HD. Key frontostriatal regions subserving goal‐directed behavior are implicated, notably the medial prefrontal cortex, anterior cingulate cortex, and ventral striatum. (**C**) Crucial neuromodulators implicated in goal‐directed behavior. Although simplex sigillum veri (simplicity is a sign of truth), this simplistic Venn diagram does not capture the complexities of goal‐directed behavior neuromodulation. Here we identify the predominant neuromodulatory system driving each cognitive process based on current animal models and human studies of goal‐directed behavior, bearing in mind their multiplex interdependence. Identification of the specific cognitive processes that are disrupted in apathy and/or impulsivity is key to developing therapeutic interventions. ACC, anterior cingulate cortex; BA, Brodmann area; dACC, dorsal anterior cingulate cortex; L, left; PFC, prefrontal cortex; pMCC, posterior midcingulate cortex; R, right; SMA, supplementary motor area; vmPFC, ventromedial prefrontal cortex; VTA, ventral tegmental area; VS, ventral striatum. (A) Adapted with permission from Le Heron et al.^49^ (B) Reprinted with permission from Martínez‐Horta et al.^50^ [Color figure can be viewed at wileyonlinelibrary.com]

Accumulating evidence, both from animal models and human work, points to key brain regions within frontostriatal circuits as crucial for goal‐directed behavior (for a review, see Bailey et al^46^). These include regions within the midbrain, the medial frontal cortex, and subcortically, the ventral striatum (which includes the nucleus accumbens).^47^ Despite marked overlap, some variability remains, with good evidence that reward and cost information are represented separately before being integrated into an overall signal that drives behavior^48^ (Fig. 2A). This crucial step is thought to occur particularly within the dorsal anterior cingulate cortex.^37^, ^38^ However, the value of alternate options (what else you could be doing) is also actively represented here, a signal thought vital to driving persistence toward goals.^51^ In such a way, although the multiple phases of normal motivated behavior can be dissociated both behaviorally and physiologically, there is a common anatomical substrate within which these networks are embedded. Disruptions to these networks have been associated with apathy and impulsivity in HD (Fig. 2B), as well as PD, Alzheimer's disease, stroke, traumatic brain injury, and others, via many neuroimaging techniques.^37^, ^52^, ^53^, ^54^, ^55^, ^56^

## Apathy in HD Is Associated With Disruption to These Same Brain Regions

Apathy in HD is associated with decreased gray matter volume and altered brain metabolism in frontal (anterior and dorsal anterior cingulate cortex and anterior insula) and subcortical regions (the ventral striatum, dorsal striatum, amygdala, and hippocampus).^50^, ^57^ Furthermore, atrophy of the middle cingulate cortex is predictive of apathy severity over time^58^ (Fig. 2B). Disruption of the white matter tracts that connect these brain regions, in particular the frontostriatal tract and uncinate fasciculus, as well as the gyrus rectus (a white matter area within the ventromedial prefrontal cortex), is also associated with apathy in HD.^59^, ^60^ There is also some evidence of altered functional connectivity between the ventral prefrontal cortex and ventral striatum associated with altered cognitive flexibility (key for adaptive decision making) in HD far‐from‐onset young adults, although more work is required to understand the functional connectivity changes associated with both apathy and impulsivity in HD.^61^

There are discrepancies between imaging studies examining apathy in HD (eg, see Baake et al,^62^ Scahill et al,^63^ and Gregory et al^64^). Aside from imaging techniques, this may relate to differing apathy measurements, variations in disease stage, and the likelihood that different mechanisms underlying apathy (as outlined earlier) may have distinct neural signatures. However, it is clear across modalities that the key frontal and striatal regions underpinning normal motivated behavior are altered in people with HD with apathy. Furthermore, a rapidly expanding literature has found markedly similar anatomical correlates of apathy across a broad range of neurological and psychiatric diseases.^37^

## Neural Correlates of Impulsivity and Apathy in HD Overlap

In contrast with apathy, to date there is a paucity of imaging studies specifically investigating the neural substrates of impulsivity in HD. This relates in part to the poor characterization of impulsivity in this condition.^11^ However, the handful of existing studies point to overlapping regions with those associated with apathy. Failed response inhibition in premanifest HD patients as well as disinhibition and pathological impulses in HD are associated with altered activation in very similar brain regions.^13^, ^65^ A recent study including premanifest HD patients found novelty seeking, a measure of impulsivity, was correlated with structural differences in the left thalamic pulvinar.^66^ Notably, work in PD has associated different dimensions of impulsivity with specific altered network connectivity patterns on diffusion imaging, which again correspond to regions implicated in apathy and crucial for normal goal‐directed behavior.^67^ Such characterization of impulsivity dimensions and their neural correlates remains to be investigated in people with HD.

## Neuromodulatory Systems Influencing Goal‐Directed Behavior

Like space and time, brain structures and neuromodulatory systems are inextricably bound. The phases of CBDM are influenced by a complex interplay between different neuromodulators, including dopaminergic, noradrenergic, and serotonergic systems (Fig. 2C). Importantly, the action of a given neurotransmitter can vary greatly depending on the specific receptors it acts on and the effects of other neuromodulators.^35^ This imbues the brain with significant flexibility but presents a major challenge for scientists trying to advance understanding and develop pharmacological treatments for behavioral disturbances. However, despite these difficulties, some key messages have emerged from the literature.

### Dopamine: Reward Incentivization and Learning

The mesolimbic dopaminergic system, projecting from the ventral tegmental area of the midbrain to the ventral striatum and anterior cingulate cortex, plays a crucial role across all phases of motivated behavior. This includes signaling potential rewards,^68^ maintaining behavioral vigor,^36^, ^69^ and driving learning.^70^ Animal studies clearly show that depletion of dopamine, either systemically or specifically within the ventral striatum, produces behavioral effects akin to lesions in these same regions,^39^, ^47^ in which animals are no longer as willing to exert effort to obtain rewards. High doses of dopamine stimulants in rodents with striatal lesions worsens response inhibition, an aspect of impulsivity.^71^ Likewise, on a backdrop of dopaminergic neuronal loss in PD, dopamine agonists stimulating D2/3 receptors may give rise to impulse‐control disorders.^72^ Although there is clear evidence of opposite behavioral effects from dopamine depletion and stimulation on some aspects of goal‐directed behavior, these can still be understood within the broader framework of disrupted CBDM. Indeed, the fact that changes along a single axis (dopamine) can lead to either apathy or impulsivity points to the close mechanistic links between these entities.^20^ Dopamine signaling occurs over different time scales, often referred to as phasic (fast) and tonic (slow).^73^, ^74^ Although phasic dopamine plays a crucial role in the learning process, encoding a prediction error that updates future beliefs and behavior,^70^ tonic dopaminergic activity has been strongly implicated in the maintenance and vigor of actions toward goals. These slower‐changing signals are thought to encode the background value within the environment, the value of “explore” behavior, and thus the opportunity cost of the current behavior.^75^

### Noradrenaline: Energization and Behavioral Switching

Whereas dopaminergic systems seem crucial for encoding reward information to guide actions in the world, noradrenaline, arising from locus coeruleus neurons in the rostral pons, is more explicitly linked to effort production and energization of behavior.^76^ Thus, it seems to play a crucial role in mobilizing resources after a decision to pursue a goal has been taken. However, illustrating the interconnectedness between phases of goal‐directed behavior, noradrenergic activity also signals information about effort costs (to influence the value of a potential behavioral option)^76^ and plays a role in learned expectations and beliefs about action costs.^77^ Furthermore, it modulates shifting behavior between exploitative or exploratory modes,^78^ with widespread noradrenergic activity reconfiguring brain networks to promote changes in goal‐directed behavior.^79^

### Serotonin: Persistence and Waiting

Although implicated in numerous processes of adaptive goal‐directed behavior,^80^ recent findings shed light on some nuanced functions of the serotonergic system. The exploitative mode, continuing with the current behavioral strategy as opposed to exploring alternatives, is largely mediated by the serotonergic system.^81^, ^82^ In addition, serotonin exerts influence over the mesolimbic dopaminergic system, and as such plays a role in motivating behavior toward stimuli predictive of rewards.^83^, ^84^ Indeed, in rodents, serotonin agonists increase dopamine release in the dorsomedial striatum, an area subserving response vigor,^85^ and in humans, dietary depletion of tryptophan, a serotonin precursor, results in reduced discrimination between rewards of different magnitudes.^84^ Depletion of serotonin in rodents worsens aspects of impulsivity;^86^ similarly, in humans, lower levels of serotonin are associated with increased impulsive behaviors, across most impulsivity domains.^86^ However, the effects of serotonin vary depending on which dimension of impulsivity is being examined, individual baseline impulsivity scores, and the specific serotonergic receptor being targeted, illustrating the inherent intricacies of this widespread neuromodulatory system.

## Neuromodulatory Systems Are Disrupted in HD

These same neuromodulatory systems key to driving motivated behavior are altered in HD.^87^ Dopaminergic dysfunction is a hallmark of HD, including altered dopamine release and receptor binding, with dopamine levels showing a biphasic profile initially increasing early in the disease course and reducing with disease progression.^88^ Noradrenergic^89^ and serotonergic^90^, ^91^ abnormalities in the striatum are evident in postmortem HD brain tissue. These changes have not yet been linked to specific behavioral impairments, but the importance of these and other neurotransmitter systems for normal behavior suggests they play a crucial role in the evolution of behavioral disorders seen in HD. As *in vivo* techniques evolve, an important future step will be linking these neuromodulatory abnormalities with breakdown in specific cognitive processes underpinning motivated behavior.

## Behavioral Studies in HD: Evidence of Disrupted CBDM

Although numerous studies have examined a range of cognitive and behavioral aspects in HD, relatively few have probed mechanisms underlying apathy or impulsivity. In this section, we interpret relevant studies within the framework of CBDM. We aim to demonstrate how application of this framework can contextualize existing work, and highlight unanswered questions. Where appropriate, we mention key results from application of this framework in other neurodegenerative diseases.

### Choice: Is It Worth It?

The integration of rewards and costs to initiate behavior is a crucial phase of goal‐directed behavior (Fig. 1). Decision‐making tasks systematically vary levels of reward and effort; computational models of choice data then disentangle the influence of each on behavior. A recent study found premanifest HD and healthy control performance did not differ with respect to reward sensitivity. However, no participants in this study were apathetic or impulsive, limiting the applicability of these findings here.^92^ Interestingly, decreased ventral striatum activation for both reward and punishment anticipation is evident in early HD,^93^ suggesting disruption to the common valuation system. However, further work is required before definitive conclusions on reward sensitivity can be drawn in HD. In contrast with HD, decreased reward sensitivity has been clearly demonstrated in apathetic patients with PD, cerebral small vessel disease, and depression,^94^, ^95^, ^96^, ^97^, ^98^ while impulsivity^67^ and impulse‐control disorder in PD are associated with increased reward sensitivity.^99^ Although these findings may superficially point to a more traditional view of apathy and impulsivity occupying opposite ends of a reward‐based spectrum, the crucial point, we argue, is the common disruption of reward processing. Although there may appear to be bias in a specific direction, the actual behavioral manifestation may depend to a large extent on the context (eg, timing of rewards and costs in relation to each other; whether the environment is stable or labile) and how reward is integrated with other decision variables. For example, buying an expensive coat now (immediately rewarding) may come at the cost of a budget deficit later; this can be considered an “impulsive” purchase (the delayed cost of financial trouble is greatly devalued). In contrast, the effort cost of washing the dishes now may lead to procrastination, or simply never bothering to do it, akin to apathy (the delayed reward of a clean kitchen is greatly devalued). This may explain in part why the same person may seem both less sensitive and more sensitive to rewards (and costs). Although this may hold true in real life, where people's decisions take place in a myriad of contexts, traditional laboratory decision‐making paradigms tend to probe only particular aspects of reward processing and costs in a single context and generally have not examined how changes in these parameters relate to apathy and impulsivity in the same person. This represents a crucial next step for the HD field.

**FIG 3:**
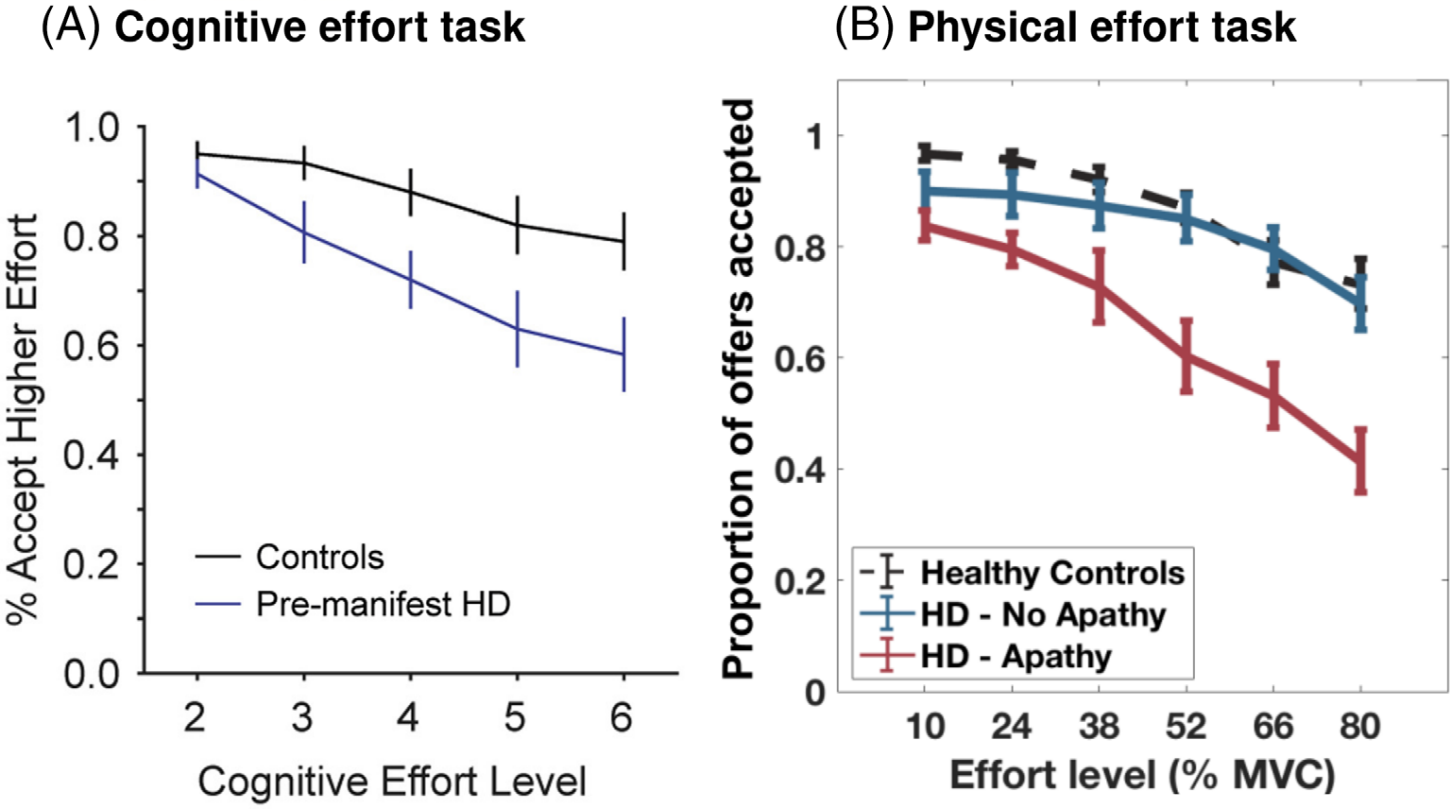
Effort hypersensitivity in Huntington's disease. (**A**) Choice data from a cognitively effortful task performed by premanifest HD participants (n = 20), none of whom had behavioral impairments, demonstrating reduced acceptance of higher effort options as effort level increased compared with controls. (**B**) A similar pattern of reduced acceptance of rewarding offers at higher physical effort levels was seen in patients with early‐stage HD (n = 18), but only in those who were apathetic. MVC, maximal voluntary contraction. (A) Reprinted with permission from Atkins et al.^92^ (B) From Le Heron et al, unpublished data. See Le Heron et al^96^ for paradigm description. [Color figure can be viewed at wileyonlinelibrary.com]

Although increased sensitivity to effort costs is not evident in the decisions of apathetic patients with PD, some evidence suggests it may be an important change in apathy occurring in HD. For example, as physical effort levels increased, HD participants with apathy were less willing to exert effort, across all reward levels, compared with those without apathy and healthy control subjects (Le Heron et al, unpublished data; Fig. 3B). Similar to this, premanifest HD participants were less willing than healthy controls to exert increasing levels of cognitive effort for reward^92^ (Fig. 3A). Effort hypersensitivity has also been associated with apathy in schizophrenia.^27^, ^31^, ^100^

Time is another important cost, and it has been suggested that impulsive individuals experience time as a greater cost than less impulsive persons,^101^ leading to choice behavior favoring the immediately available option. Indeed, impulsivity in drug addiction and substance abuse, as well as PD, is associated with steep delay discounting (favoring immediate rewards vs. larger rewards available later) in decision‐making paradigms.^17^, ^102^, ^103^ In transgenic HD rodent models, steep delay discounting is also evident compared with wild‐type rodents.^104^, ^105^ A study examining delay discounting choices in HD found evidence of impulsive choices, although half of the participants had inconsistent preferences, making the choice data difficult to interpret.^106^ This warrants replication, given that the delay discounting task has been used successfully in participants with PD,^107^, ^108^ frontotemporal dementia, and Alzheimer's disease.^109^, ^110^ However, there is also evidence that time perception is altered in HD, including decreased accuracy and precision (greater variability) in time production,^111^ time discrimination,^112^ and time estimation tasks.^113^, ^114^ This increased variability suggests increased *noise* in decisions made by people with HD related to time, which may explain the inconsistent preferences of people with HD in the delay discounting task cited earlier. It could also mean that an apparently pathological *impulsive* preference for immediate rewards is actually an adaptive behavioral response to a valuation system that cannot accurately predict future rewarding states or compute the background reward rate of an environment.^115^

Increased risk taking characterizes certain forms of impulsivity and has been associated with PD^17^ and substance abuse.^116^ Similarly, HD participants make more high‐risk/high‐reward choices^117^ and place a greater number of higher bets^118^ compared with controls, suggesting that risk devalues rewards differently in HD. Decreased sensitivity to loss (the negative outcome of these “riskier” decisions) in HD has been demonstrated on an autonomic level,^119^ in functional imaging^93^ and behaviorally.^120^ This raises the intriguing possibility that risky choices may stem from an underweighting of potential losses associated with the choice. Such loss insensitivity may also generalize to a reduced sensitivity to the opportunity cost of actions (ie, the loss associated with unchosen options, intimately linked to the background reward rate of an environment). Of interest, healthy people tend to exert more effort to avoid loss than to ensure gain,^121^, ^122^ where *loss* is conceptualized as a negative reinforcer, or punishment. Could loss insensitivity in HD lead to diminished effort exertion to avoid loss? This has yet to be examined.

### Persistence and Vigor: Is It Still Worth It?

Although persisting to achieve a chosen goal remains a hallmark of goal‐directed behavior, the vigor with which a goal is pursued and the ability to switch actions if a better option presents itself are also closely related components of CBDM that shape motivated behavior. No studies have directly examined persistence and vigor in the context of changing environmental reward rates in HD, although one recent study investigated persistence. Participants engaged in a virtual car race against a computerized opponent. Controlling for motor impairment, apathy in HD was associated with prolonged duration of engagement compared with controls.^120^ However, studies examining these processes, particularly within the context of HD, must be able to dissociate putative effects from simpler phenomena, such as motor impersistence or perseveration.

Healthy human participants tend to explore alternate options in rich environments but exploit current options in poor environments.^123^ In other words, choice behavior is dramatically altered by the estimation of background reward rate. This may be impaired in HD, for example, by increased noise in time perception,^111^, ^112^ leading to unreliable estimations of the opportunity cost of time, a key decision variable that guides the consequent vigor with which actions are pursued.^69^ Paradigms to investigate this phase of decision making have recently been developed^75^ but have not yet been applied to apathy or impulsivity in HD.

### Learning: Was It Worth It?

Broadly, past experience modulates future behavior. Adaptive behavior thus depends on the ability to update values (of costs and rewards) from past outcomes. Both apathy and impulsivity may arise from disruptions to this learning process; indeed, numerous studies point to altered learning in HD.

HD participants have higher learning rates (the extent that prediction errors alter future behavior)^124^, ^125^ compared with controls. Interestingly, this may be specific for positive feedback, indicating “overlearning” from rewards, whereas learning from loss occurs at a slower rate.^126^ This same pattern is evident neurally, with increased striatal response to reward‐predicting cues compared with loss‐predicting cues in a functional magnetic resonance imaging study.^127^ Similarly, apathy in HD has been associated with deficits in instrumental learning, particularly after large losses, although less so after large rewards.^120^ Thus, current evidence suggests that learning is altered in HD specifically according to *stimulus valence* (reward vs. loss). Although debated, there is evidence for a common valuation system encoded in medial frontal and ventral striatal regions.^30^, ^32^ In this light, learning on either end of this valuation spectrum is affected in HD (see Frank et al^128^ for a computational account of dopamine function and this phenomenon), although further work is needed to understand how this relates to both apathy and impulsivity.

## Confounding Factors

It is important to note that in HD, apathy and impulsivity may occur amidst a myriad of cognitive and psychiatric impairments. These include, among others, impaired attention and set‐shifting (cognitive skills key for adaptive behavior) and depression, anxiety, and irritability (common mood disorders in HD).^6^, ^129^ Both mood and cognition can also influence goal‐directed behavior. For example, heightened emotional arousal, independent of motivational state, increases effort production in incentive motivation tasks,^130^ while aspects of depression are associated with altered reward processing.^98^ Thus, future work within the CBDM framework must account for these variables, as well as others, such as motor function.

## Treatment of Apathy and Impulsivity Informed by a CBDM Framework

Efficacious therapies for apathy and impulsivity are lacking. As outlined in this review, the behavioral phenotypes of each are composed of various subcomponents or processes (Fig. 1), each of which are to some extent driven by different neuromodulatory systems (Fig. 2C). Thus, a crucial goal for the field is to identify robust methods that index these dissociable processes in individual people with HD who have apathy and/or impulsivity. As an example, for apathy driven by poor reward incentivization, or in which background environmental rewards are undervalued, the dopaminergic system may be a key pharmacological target. In contrast, the noradrenergic system may be a key target for apathy associated with hypersensitivity to effort costs, to improve both evaluation of effort costs and actual exertion of the effort. Noradrenaline may also be key to reducing impulsivity, given that the selective noradrenergic reuptake inhibitor, atomoxetine, has been found to improve multiple dimensions of impulsivity in rodents, and similarly is routinely used in the treatment of attention deficit hyperactivity disorder.^131^

Despite these biological rationales, trials in HD have not demonstrated convincing results to date. Bupropion, a noradrenaline and dopamine reuptake inhibitor, failed to improve apathy in HD.^132^ Similarly, modafinil, a dopamine reuptake inhibitor (among other actions), had no beneficial effects on cognition or mood in 22 patients with mild HD.^133^ A randomized controlled trial of atomoxetine failed to improve attention, psychiatric function, or executive function in 20 patients with mild HD.^134^ Notably, however, a retrospective analysis of the TRACK‐HD data did find that use of selective serotonergic or noradrenergic reuptake inhibitors was associated with improved apathy and total behavior scores, after adjusting for confounding variables.^135^ Similar treatment strategies for apathy have been suggested in PD,^136^ and in this setting some evidence does exist for favorable effects of dopamine agonists^137^, ^138^ and cholinesterase inhibitors,^139^ albeit without clear evidence of the mechanisms underlying their efficacy (see Liu et al^140^ for a review). Furthermore, the 5‐HT2C partial agonist agomelatine has been found to improve apathy in people with frontotemporal lobar degeneration, another condition in which apathy and impulsivity frequently co‐occur.^141^ Future drug trials should include specific behavioral paradigms that index the phases of motivated behavior being targeted in addition to broad outcomes, such as questionnaires and clinical scales. Indeed, behavioral parameters of different phases of goal‐directed behavior could be used to preselect patients most likely to benefit from a given pharmacological intervention.

To complicate matters, current treatments used in HD also alter these neuromodulatory systems. In rodent studies, tetrabenazine biases behavior toward “low‐effort” options,^142^, ^143^ mirroring the phenotype of apathy, while in HD, antipsychotic use has been associated with worsening apathy.^144^ Interestingly, however, an analysis of the Enroll‐HD data found antidopaminergic medication usage was not associated with worsening apathy.^145^ Thus, on the backdrop of HD pathology, the influence of dopaminergic drugs on behavior remains unclear. Furthermore, heterogeneity in clinical response to neuromodulators exists both across and within individuals.^146^ Recent intriguing work highlights the importance of the *baseline state of target regions* for neuromodulator efficacy. This is consistent with the inverted‐U shape dose–response curve apparent across monoaminergic and cholinergic systems.^147^ For instance, response to atomoxetine to improve response inhibition in PD depended on locus coeruleus integrity.^148^ Determining the baseline state of key nuclei may also help with stratified patient selection for future behavioral drug trials in HD. All this points to the importance of individualizing patient treatments, an approach that can occur only with proper understanding of normal pathways and appropriate techniques to index these. A mechanistic understanding of apathy and impulsivity would also advance the development of tailored nonpharmacological/behavioral therapies, which currently do not have a strong evidence base. Finally, given the impact of these traits on those closest to the person with HD, the value of caregiver education cannot be overstated, not least to aid understanding of these often challenging behaviors.

## Future Research Directions

Although the neuroscientific community has made significant advances in understanding goal‐directed behavior in healthy individuals, these findings are only just beginning to be applied to problems such as apathy and impulsivity in HD. We have embedded current neural and behavioral work in HD within the neurobiologically grounded framework of goal‐directed behavior and, as we have highlighted in relevant sections throughout this review, many outstanding research questions remain (Table 2). In addition, future work should also consider the proposed dimensions of apathy and impulsivity (in relation to the decision‐making framework) and the stage of HD, as it is plausible that different mechanisms could drive behavioral changes as the landscape of pathology evolves over time.

**TABLE 2:** Outstanding research questions

Which dimensions of apathy and impulsivity overlap or are dissociable in HD?
What is the trajectory of co‐occurring apathy and impulsivity in HD?
What are the dissociable contributions of reward sensitivity and effort sensitivity in abnormal motivated behavior in HD?
Is background reward estimation altered in HD?
How is time perceived in persons with apathy and impulsivity in HD?
How does altered learning influence future valuation and subsequent behavior in HD in those with co‐occurring apathy and impulsivity?
Which neuromodulators and brain networks underlie apathy and impulsivity in HD, and how do these brain networks change over the disease course?
How can drug therapies be optimized to target specific components of apathy and impulsivity in HD, working toward personalized medicine?

## Conclusions

Understanding the way people move ‐ a normative model of motor function ‐ has been key to delineating the mechanisms involved in disordered movement. Identifying where breakdown occurred and attempting to ameliorate this has led to the development of various movement disorder therapies, both pharmacological and physical.^149^ But how are people typically motivated? What are the mechanisms affected in people with disordered behavior? As we advanced in this review, such a normative framework of goal‐directed behavior has recently been crystallized. Work in neurological and psychiatric disease using this framework has met with much success in identifying *which mechanisms* are altered. Many valuable insights stand to be gained from its application to HD to uncover, and ultimately treat, the altered brain mechanisms underlying apathy and impulsivity in this condition.

## Author Roles

L.M., C.L.H., and C.O. wrote and edited the final version of the manuscript.

## Full Financial Disclosures for the Previous 12 Months

L.M. is supported by a University of Otago doctoral scholarship. C.O. is supported by a Talented Researcher Scheme Fellowship from the Faculty of Medicine and Health, University of Sydney. C.L.H. is employed by the Canterbury District Health Board. He has received research funding from the Canterbury Medical Research Foundation and Pacific Radiology Group Research and Education Trust.

## Data Availability

Data sharing is not applicable to this article as no new data were created or analyzed in this study.
